# Facial cutaneous lesions of dental origin: A case series emphasizing the awareness of the entity and its medico-legal consequences

**DOI:** 10.1016/j.ijscr.2018.10.032

**Published:** 2018-10-26

**Authors:** Mohammad M. Al-Qattan, Muhammed I. Almotairi

**Affiliations:** a Division of Plastic Surgery at King Saud University, Riyadh, Saudi Arabia; b Division of Plastic Surgery at King Faisal Specialist Hospital and Research Center, Saudi Arabia

**Keywords:** Facial lesions, Skin sinus, Dental origin, Medico-legal, Dental root infection

## Abstract

**Introduction:**

Facial cutaneous lesions of dental origin are rare and hence the correct diagnosis is usually missed on the initial presentation to the surgeon. We present the largest series in the surgical literature to increase the awareness to this entity and emphasize that the wrong diagnosis may lead to medico-legal claims.

**Patients and methods:**

This is a retrospective study of 28 patients seen between 1994 and 2017.

**Results:**

The series had one child and 27 adults. The presentation varied and included: a nodule, a subcutaneous soft mass, a sinus, an abscess, a cyst, a pigmented skin lesion and a scar. Thirteen cases had an initial wrong diagnosis and of these 4 had surgery to the lesion. Two out of the 4 surgically treated lesions ended with medico-legal claims.

**Conclusions:**

The surgeon should be aware of this rare entity and have a high index of suspicion. Dental treatment is curative and there is no need to operate the facial lesion. The wrong diagnosis and unnecessary surgery may lead to medico-legal claims.

## Highlights

• Facial lesions of dental origin are rare.
• We present the largest series in the surgical literature.
• The presentation is variable and dental treatment is curative.
• The wrong diagnosis/treatment may lead to medico-legal claims.

## 1 Introduction

Facial cutaneous lesions of dental origin are formed because of underlying dental root infection and subsequent sinus formation towards the face [1]. They are rare and hence the correct diagnosis is usually missed on the initial presentation to the surgeon [1]. Most case reports and all large case series were published in the dental and dermatology literature [[1], [2], [3], [4], [5]]. Furthermore, none of the previous reports stressed on the medico-legal consequences of the wrong diagnosis and treatment.

The aims of the current report are: to present the largest series in the surgical literature of facial cutaneous lesions of dental origin, increase the awareness of plastic surgeons to this rare entity, and document that the initial wrong diagnosis/treatment may lead to medico-legal consequences. The case series is compliant with the PROCESS guidelines [6].

## 2 Material and methods

This is a retrospective study of all patients with facial cutaneous lesions of dental origin who presented to the senior author (MMA) between 1994 and 2017. The following data were collected: age, sex, origin and location of the cutaneous lesion, the clinical appearance of the lesions, the initial diagnosis made, investigations done to reach the correct diagnosis, the treatment and its outcome, and finally documentation of any medico-legal complaints.

## 3 Results

A total of 28 patients were reviewed. There was a ten-year-old child; and the remaining 27 were adults (mean age of 30 years, range: 18–49 years). There were 14 males and 14 females.

In 12 cases, the facial lesion originated from the maxillary teeth: 11 were located in the cheek and 1 below the lateral canthus of the eye. The remaining 16 lesions arose from the mandibular teeth; and their locations were more variable: along the mandibular body in 6, at the mandibular angle in 4, the submandibular area in 4, and the chin area in 2.

The most common clinical appearance of the lesion was a non-tender nodule (n = 8) followed by diffuse non-tender subcutaneous soft mass with overlying skin hyperpigmentation (n = 6). Other clinical presentations included: A discharging sinus (n = 4), an acute abscess (n = 4), a nodulo-ulcerative lesion (n = 2), a cystic swelling mimicking a sebaceous cyst (n = 2), a pigmented skin lesion (n = 1), and a hypertrophic scar (n = 1). In the latter presentation, there was a history of a discharging sinus that healed into a scar.

**Fig. 1:**
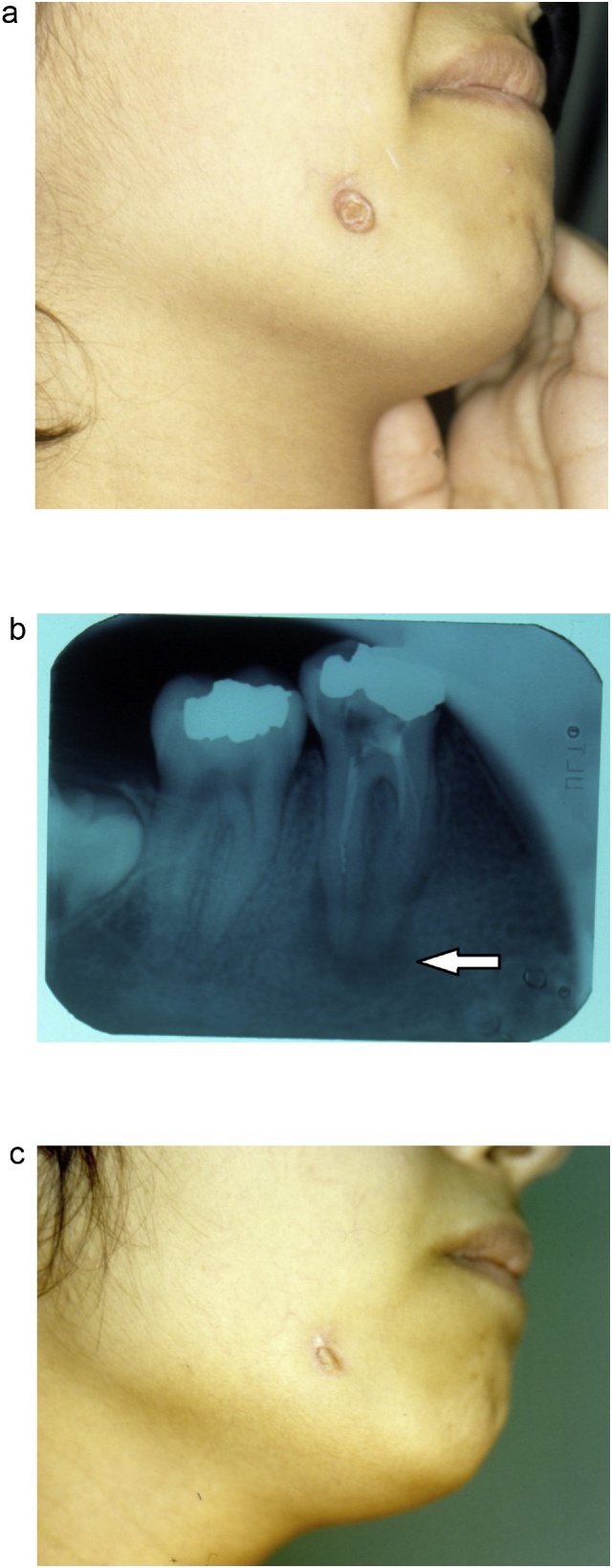
a) A nodulo-ulcerative lesion along the mandibular border. There was an adjacent teeth caries. b) Dental x-rays showing the radiolucency around the chronically infected tooth root (arrow). c) Resolution of the ulcer following dental extraction.

Out of the total 28 cases, 15 patients first presented to the senior author. The dental origin was suspected in all patients at initial presentation. This suspicion was based on the presence of dental caries near the skin lesion as well as the palpation of a submucosal cord-like structure under the buccal mucosa extending from the alveolar margin near the affected tooth towards the skin lesion. All patients were referred to the dental department and the correct diagnosis was confirmed radiologically by the presence of radiolucency around the chronically infected tooth root. Dental treatment (tooth extraction) was curative in all cases (Fig. 1).

The remaining 13 patients initially presented outside the senior author’s clinic and these were all wrongly diagnosed as either skin tumor or a sebaceous cyst. Of these 13 cases, 4 cases underwent surgery at the initial presentation (outside our clinic) as follows: Incision and drainage of an “infected sebaceous cyst” (n = 1), incisional biopsy of a “benign skin tumor” (n = 1), and excisional biopsy of a skin mass/cyst (n = 2). The pathology report of the latter 3 cases was a “chronic inflammatory mass”. Upon presentation to our clinic, the correct diagnosis was reached (at our clinic) in all cases and dental treatment was curative.

**Fig. 2:**
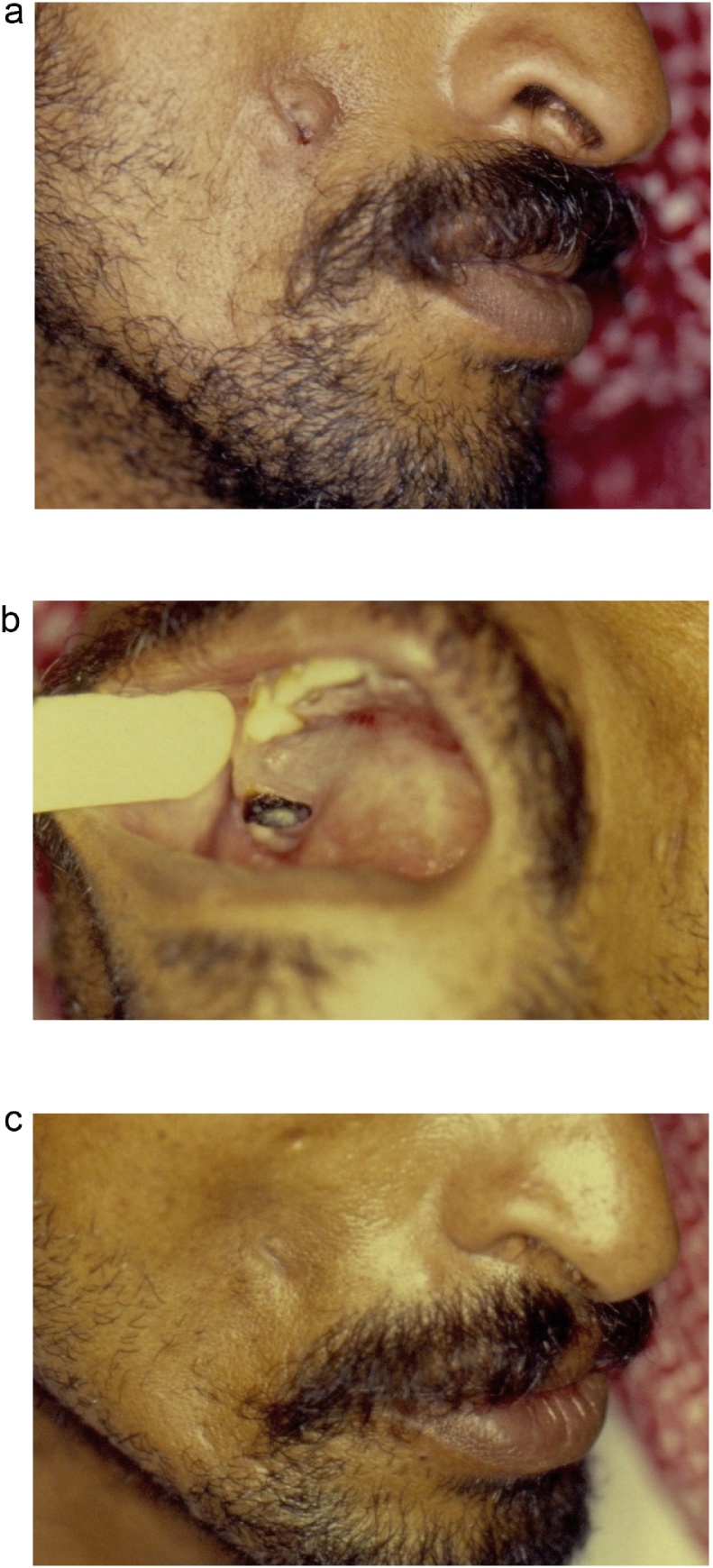
A case treated outside our clinic with incisional biopsy from a “skin nodule”. a) The appearance at the time of presentation to our clinic. Note the scar from the incisional biopsy in the center of the nodule. b) Teeth caries. c) The resolution of the nodule after dental treatment. The remaining scar was considered as an “unnecessary” scar; but it did not result in a medico-legal claim.

Two out of the 4 operated cases did not result in medico-legal claims. The first case underwent incision and drainage of an “infected cyst” at a local hospital. This resulted in a scar but the patient was informed that the abscess required drainage regardless of the diagnosis. The second case underwent (at a local hospital) incisional biopsy of a “benign skin tumor” which also resulted in a linear but acceptable scar (following the completion of the dental treatment); and no medico-legal claims were filed (Fig. 2).

**Fig. 3:**
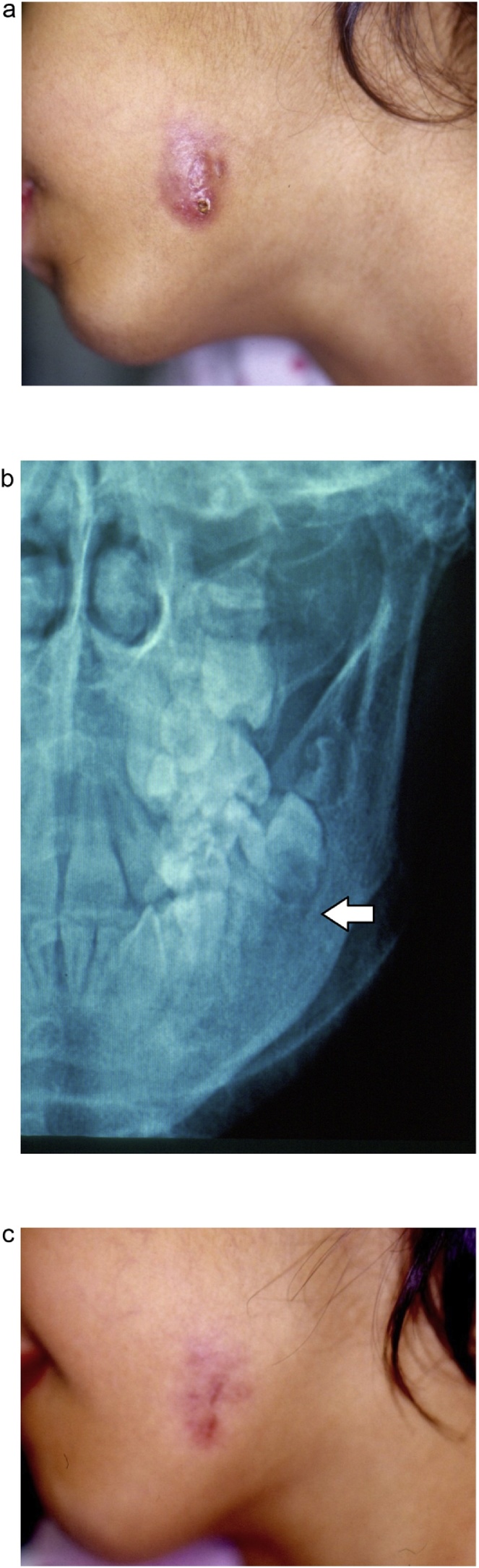
A case treated outside our clinic with excisional biopsy of the skin lesion. One month later, the patient was referred to our clinic with recurrence. a) The appearance at the time of presentation to our clinic. There is recurrence with chronic inflammation at the site of the scar. b) X-ray showing the radiolucency of the adjacent molar tooth (arrow). c) The resolution of the chronic infection after dental treatment. The remaining scar was long and was considered “unnecessary”, resulting in a medico-legal claim.

The remaining 2 cases that underwent surgery at their local hospitals ended with medico-legal claims. These two cases were from the second group of patients who had an initial wrong diagnosis and surgery for the lesion at initial presentation to their local hospitals. Both patients underwent excisional biopsy of a ‘facial mass/cyst’, resulting in a long scar (following the completion of the dental treatment). Medico-legal claims were filed in both cases and both claims ended in favor of the patient against the surgeon (Fig. 3).

## 4 Discussion

Our case series of facial skin lesions of dental origin is the largest one in the surgical literature and aims to increase the awareness of surgeons to this rare entity. Furthermore, we bring the attention that the wrong diagnosis/treatment may result in medico-legal claims.

The pathophysiology of facial lesions of dental origin is well described in the literature [7]. The infection from the root of the tooth spreads through the path of least resistance, perforating the outer cortex of the mandible or maxilla. The path in the soft tissues is decided by facial muscle attachments. Most sinuses in adults pass within the muscle attachments (the buccinator, the myelohyiod, and the masseter muscles); and this results in a sinus that opens intra-orally. In these cases, the diagnosis is easily made. In adults the path of the sinus is rarely above or below these muscle attachments; resulting in an extra- oral facial lesion or sinus [7]. In children, the alveolar process is not fully developed and the incompletely erupted infected tooth is more deeply seated. Hence, there is greater likelihood for the sinus to go beyond the muscle attachment; presenting extra-orally as a facial lesion [8].

Our case series aims to increase the awareness of the variable presentation of facial lesions of dental origin; and we stress on the fact that a classic discharging sinus is not the most common presentation. The surgeon should also be aware of unusual presentations of such lesions and these include: midline upper lip and chin lesions [7], bilateral facial lesions [9], a facial lesion of dental origin in an edentulous patient (arising from an embedded tooth fragment) [10]; and lesions mimicking basal cell carcinoma [11].

The correct diagnosis is only made if the surgeon is aware of the entity and also if there is a high index of suspicion. The senior author always rules out the dental origin in every patient presenting with a facial lesion. The diagnosis is suspected if there is teeth caries and the palpation of a cord-like structure connecting the skin lesion to the alveolar ridge. The diagnosis is then confirmed by dental x-rays. Dental treatment is curative. Finally, our report emphasizes that the wrong diagnosis/treatment may lead to medico-legal claims.

## 5 Conclusions

Facial lesions of dental origin are rare and most case reports and all large case series have been reported in the dental and dermatology literature. We present the largest series in the surgical literature and show that the presentation is variable. The correct diagnosis is only made if the surgeon is aware of the entity. Dental treatment is curative and there is no need to operate on the facial lesion itself. Finally, we emphasize that the wrong diagnosis/treatment may lead to medico-legal claims.

## Conflict of interest

None.

## Funding

The work was supported by the College of Medicine Research Center, Deanship of Scientific Research, King Saud University, Riyadh, Saudi Arabia.

## Ethical approval

The study was approved by the research committee, National Hospital (Care), Riyadh, Saudi Arabia.

## Consent

Written informed consent was obtained from adult patients as well as from the parent of the patient who is under 16 for publication. A copy of the written consent is available for review by the Editor-In-Chief of this journal on request.

## Author’s contribution

The senior author (MMA) performed and managed all patients. Both authors collected the data, did the literature review, and wrote the manuscript.

## Registration of research studies

Research registry UIN: 4083.

## Guarantor

M M Al-Qattan.
